# A successful case of preimplantation genetic testing for monogenic disorder for aplasia cutis congenita

**DOI:** 10.3389/fped.2022.1005272

**Published:** 2022-11-15

**Authors:** Xiu-Fang Yang, Shang-Wen Shi, Yun Ye, Kang Chen

**Affiliations:** ^1^ Department of Neonatology, Zhongshan Hospital Affiliated to Sun Yat-Sen University, Zhongshan, China; ^2^ Reproductive Medicine Center, Zhongshan Hospital Affiliated to Sun Yat-Sen University, Zhongshan, China; ^3^ Molecular Inspection Center, Zhongshan Hospital Affiliated to Sun Yat-Sen University, Zhongshan, China

**Keywords:** neonates, aplasia cutis congenita, ITGB4 gene, case report, preimplantation genetic testing for monogenic disorders (PGT-M)

## Abstract

**Background:**

Aplasia cutis congenita (ACC), also called congenital cutaneous hypoplasia, is a serious disease in newborns. Children with ACC often die due to wound infections and bleeding. How the incidence of ACC can be reduced is a question that needs to be solved urgently.

**Case report:**

We reported a mother who had delivered two children with ACC, both of whom were diagnosed with ACC type VI, skin defects, limb deformities, and congenital heart malformations. One infant died a few days after birth, and another died *in utero* in the second trimester. Genetic testing in both children showed a heterozygous mutation in the *ITGB4* gene [17q25 exon 8, c. 794 dupC, (*p*. Ala266fs) and exon 15, c. 1860G > A]. The mother later successfully gave birth to a healthy baby using Preimplantation Genetic Testing for Monogenic disorders(PGD-M).

**Conclusion:**

The PGD-M technique is highly valuable in reducing the incidence of ACC and improving the prognoses of newborns.

## Introduction

Aplasia cutis congenita (ACC), also known as congenital skin hypoplasia, refers to the hereditary loss of a newborn's skin, dermis, and even subcutaneous tissues (including muscles and bones). Approximately 500 ACC cases have been reported since its first description by Cordon in 1,767 (1). It is a rare clinical disease with a low incidence of about 1/100 000–3/10 000 (2, 3). Neonates with ACC may have other systemic malformations. The pathogenesis of ACC is still unclear, but it is thought it may be related to genetic factors, congenital uterus malformations, fetal skin adhesion to the amniotic membrane, teratogenic drugs in early pregnancy, or viral infection (4, 5). In this study, we present a family with a mother who had previously given birth to two newborns with ACC that died but then successfully gave birth to a healthy child after using Preimplantation Genetic Testing for Monogenic disorders (PGT-M).

## Patients, clinical report, and results

**Figure 1:**
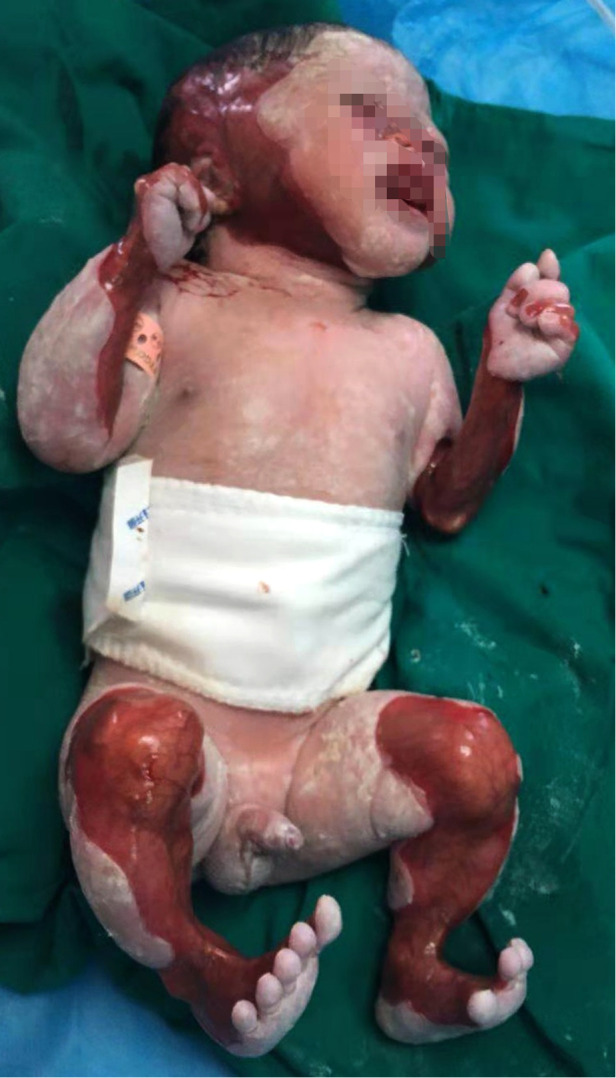
The first child of this mother with aplasia cutis congenita.

We reported that a 27-year-old mother's first child was a male baby born after 37 weeks of pregnancy in the Obstetrics Department of Zhongshan Hospital, Affiliated with Sun Yat-Sen University (also known as Zhongshan City People's Hospital). After birth, he was admitted to the department of neonatology due to partial skin defects, deformities, and shortness of breath. The weight at birth was 2.48 kg, and the body length was 46 cm. The mother had a history of gestational diabetes mellitus and premature rupture of membranes. Herpes simplex virus, varicella-zoster virus, cytomegalovirus, and rubella virus infections were excluded. At birth, the Apgar score (an evaluation of how well the baby tolerated the birth process) was 9–10–10. The mother complained of acute nephritis in her childhood and had previously had two miscarriages. This baby was categorized as ACC type VI, which is associated with epidermolysis bullosa (EB) on the face, head, and limbs. The skin defect accounted for 30% of his body surface. Abnormalities of the nose and limbs accompanied the bullae on the skin and mucosa. He did not suffer from esophageal atresia, esophageal lesions, or pyloric atresia. His left arm was significantly smaller than the right because of muscular dysplasia (Figure 1). The peripheral blood white blood cell count reached 12,670 cells/mm^3^, and his blood gas indicated metabolic acidosis with a lactic acid (LAC) level of 5.27 mmol/L. The results of serum *C*-reactive protein, blood ammonia biochemistry, and hepatobiliary biochemistry tests were normal. A chest x-ray revealed there was no apparent abnormalities in both lungs. Echocardiography revealed an atrial septal defect (5 mm) and a patent ductus arteriosus (3 mm). The size and function of his heart were normal. The ultrasound of the liver, spleen, kidneys, and ureters showed normal results. The treatment of these conditions post-birth for the infant included piperacillin sodium, as well as careful skincare and infection prevention. He also underwent congenital genetic metabolic disease screening, peripheral blood chromosome karyotyping, chromosomal microarray analysis (Affymetrix GeneChip® System 3000Dx v.2 Chip System, Affymetrix, United States) and whole exome sequencing (WES) (Nova Seq 6000 Genome Analyzer, Illumina, United States). Steps of WES included: extracting genomic DNA, fragmenting the extracted DNA, PCR amplification and purification, analyzing the sequencing results to obtain the suspected candidate mutations in the exon regions of 4,811 clinically relevant genes according to Human Gene Mutation Database, HGMD Professional, Online Mendelian Inheritance in Man and www.genetests.org. Finally, Sanger sequencing was used to verify the mutations, and the corresponding loci of the parents were detected.

**Figure 2:**
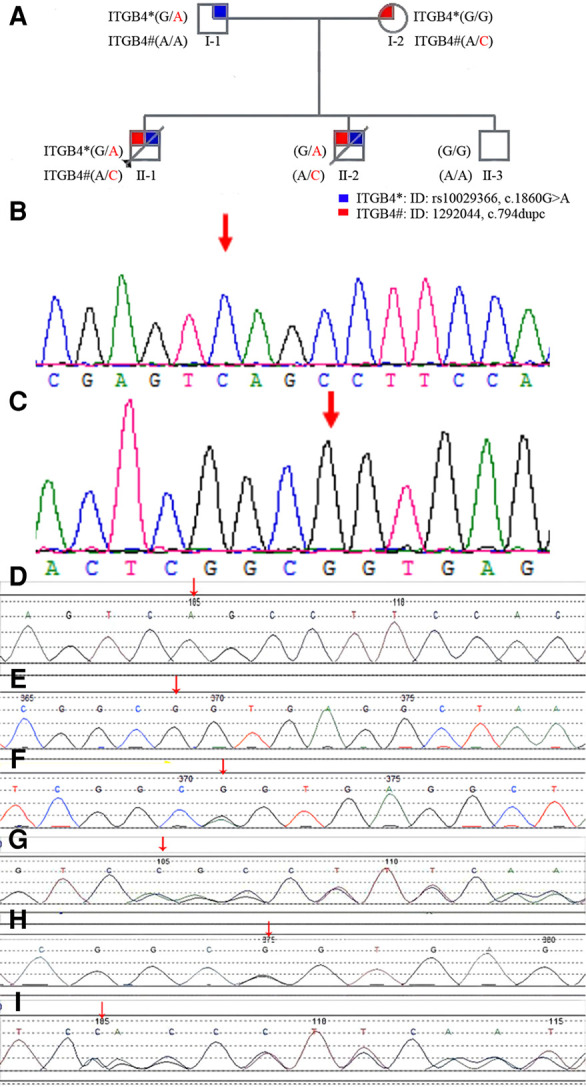
(**A**): family pedigree diagram. Shapes in blue indicate affected individual (I-1) with NM_000213.5 (*ITGB4*):*c.1860G > A.* Shapes in red indicate affected individual (I-2) with NM_000213.5 (*ITGB4*):*c.794dupC (p.Ala266fs).* Patients II-1,II-2, carried a compound heterozygous mutation with NM_000213.5 (*ITGB4*):*c.1860G > A* and NM_000213.5 (*ITGB4*):*c.794dupC (p.Ala266fs)* inherited from the parents. II-3 is a healthy boy who is unaffected with these two mutations of ITGB 4 gene. (**B**): The gene mutation in the *ITGB4* gene (*17q25 Exon 8, c.794dupC, [p.Ala266fs])* was not identified in the youngest healthy boy. (**C**): The gene mutation in the *ITGB4* gene [*17q25 Exon 15, c.1860G > A*] was not identified in the youngest healthy boy. (**D**): The gene mutation in the *ITGB4* gene (*17q25 Exon 8, c.794dupC, [p.Ala266fs*]) was not identified in the father. (**E**): The gene mutation in the *ITGB4* gene [*17q25 Exon 15, c.1860G > A*] was not identified in the mother. (**F**): The heterozygous gene mutation in the *ITGB4* gene [*17q25 Exon 15, c.1860G > A*] was identified in the father (arrows point to the site of mutation). (**G**): The heterozygous gene mutation in the *ITGB4* gene (*17q25 Exon 8, c.794dupC, [p.Ala266fs]*) was identified in the mother (arrows point to the site of mutation). (**H**): The heterozygous gene mutations in the *ITGB4* gene [*17q25 Exon 15, c.1860G > A*] were identified in the first two children of this mother (arrows point to the site of mutation). (**I**): The heterozygous gene mutations in the *ITGB4* gene (*17q25 Exon 8, c.794dupC, [p.Ala266fs]*) were identified in the first two children of this mother(arrows point to the site of mutation).

The hereditary metabolic disease screening and peripheral blood chromosome karyotyping showed no abnormality. Through whole exome sequencing, the compound heterozygote variants in the integrin, beta 4 (*ITGB4*) gene *(17q25 exon 8, c.794dupC, [p.Ala266fs], and exon 15, c.1860G > A)* were revealed in the newborn, which originated from his mother and father, respectively (Figure 2). Based on the clinical picture of the defect and the perinatal history, we diagnosed the infant as being prematurely born with low-birth weight and as having ACC type VI (using the Frieden classification system). On July 18th, 2018, the child was discharged at the family's request and died at home on July 20th, 2018.

This mother was pregnant again in December 2018. When the fetus was 11 weeks into pregnancy, a fetal chorionic biopsy and gene mutation detection with chromosomal microarray analysis, sanger sequencing and whole exome sequencing revealed that this fetus carried two heterozygous mutations of the ITGB4 gene (17q25 exon 8, c.794dupC, [*p*.Ala266fs] and exon 15, c.1860G > A). The mother was admitted to the Obstetrics Department of Zhongshan Hospital, Affiliated with Sun Yat-Sen University, on March 10th, 2019, where she was required to be placed into induced labor because the fetus died *in utero*. The total area of the skin defect of this fetus accounted for 45% of the whole body. His skin defect was also associated with EB on the face, trunk, and limbs. Due to this and his limb abnormalities, he was categorized as having ACC type VI ACC.

After these two pregnancies, the mother received genetic counseling. In July 2021, she gave birth to a healthy boy that did not have these two heterozygous mutations of the ITGB4 gene (17q25 exon 8, c.794 dupC, [*p*.Ala266fs] and exon 15, c.1860G > A). nor No skin defects through her receiving PGD-M in the Reproductive Medicine Center of the First Affiliated Hospital of the Sun Yat-sen University. The family genetic history is shown in Figure 2.

## Discussion

ACC is a rare neonatal disease. Its incidence is low and sporadic, but it also has a genetic tendency. So far, the etiology of ACC is unknown. The most common causes of ACC include fetal chromosomal or genetic abnormalities, traumas, amniotic fluid or membrane abnormalities, and intrauterine infections (6–12). The diagnosis of ACC is based on the clinical presentation of a well-defined skin defect in a region or regions of the body visible to the newborn after birth, with (or without) subcutaneous tissue (including muscle, bone) defects or hypoplasia. The shapes of the skin defects are not fixed and are often irregular. The size and depth of the skin defects vary in the individuals examined.

**Figure 3:**
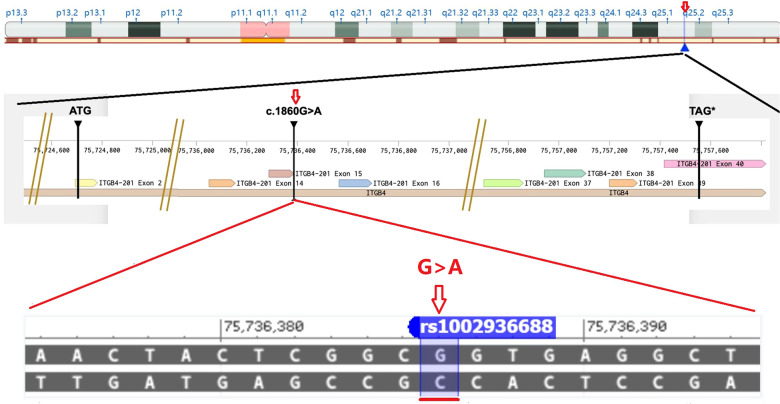
The position of *c.1860G > A* in the *ITGB4* gene at exon intron sequence resolution was showed in graphical represention.

Histopathologic examinations often show that there is a missing epidermis and/or dermis, and some or all of the fat in the subcutaneous tissue is missing (13). The disease can be accompanied by other systemic diseases or complications, such as EB, a cleft lip and palate, and polycystic kidneys (14). In 1986, Frieden (15) first classified newborn skin defects into nine categories based on their locations, characteristics, and presence of other systemic diseases, including type I: simple scalp congenital defect (scalp defect without other systemic diseases); type II: scalp defect with limb abnormalities; type III: scalp defect with an epidermal nevus; type IV: congenital skin defect with skin hypoplasia; type V: congenital skin defect with a mummified fetus; type VI: congenital skin defect with EB; type VII: limb skin defect without EB; type VIII: congenital skin defect caused by intrauterine infection; and type IX: congenital skin defect with other malformation syndromes [Patau syndrome, Setleis syndrome, Johanson–Blizzard syndrome, Goltz syndrome, ADAM complex, etc]. Scalp lesions were the most common type, accounting for about 70% of all cases of ACC (15). Currently, there is no special treatment for ACC, which would constitute general nutrition support, reasonable treatment of wound surface, active prevention, and infection control. If the wound is not treated correctly, it may affect the healing of the skin defect, prolong the course of treatment, and increase the incidence of infections and septic shocks. The treatment would depend on the wound size and whether signs of inflammation are present. The skin defect of the face and limbs of the two children referred to earlier were severe. Skin grafting could be considered after birth, which could potentially improve the prognosis. The two children with poor prognoses that were discussed earlier were associated with two heterozygous mutations of the ITGB4 gene. *ITGB4* gene is a gene encoding the integrin 4 subunit of the laminin receptor. Integrins are heterodimers composed of alpha and beta subunits, which are non-covalently related transmembrane glycoprotein receptors. Integrins mediate cell-matrix or cell-cell adhesion and transduce signals that regulate gene expression and cell growth.This bindings of ITGB4 and IGF1, IGF2 are essential for IGF1 and IGF2 signaling. Mutations in the *ITGB4* gene can cause borderline EB with pyloric atresia or non-Herlitz borderline EB, which is inherited in an autosomal recessive manner. Among its related pathways are GPCR pathway and actin nucleation by ARP-WASP Complex. NM_000213.5 (ITGB4):c.794dupC (*p*.Ala266fs) variant was reported previously in public database. The solr relevance score is 4.570 between this variant and epidermolysis bullosa. The solr relevance score of this variant and pyloric atresia, protein-losing enterophathy, junctional 5b is 0.253–0.358. NM_000213.5 (ITGB4):c.1860G > A is a synonymous variant. The position of the variant c.1860G > A is chr17:75736386 (GRCh38.p13). The frequency of this variant is 0.000008 (2/264690, TOPMED). The position of *c.1860G > A* in the *ITGB4* gene at exon intron sequence resolution was showed in graphical represention (Figure 3). This variant is no reported in ClinVar.

**Figure 4:**
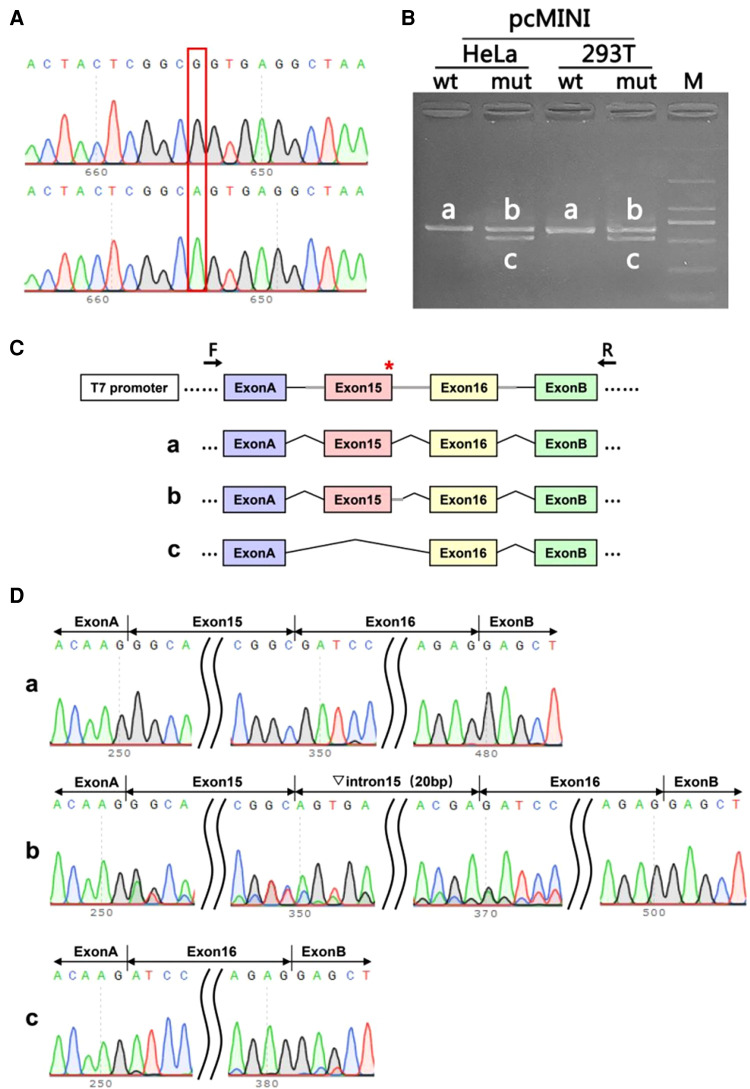
The splicing assay had been performed on mRNA obtained from the parent harboring the synonymous variant c.1860G > A. (**A**): The sequencing diagram of minigene assay, wild-type(wt) on the top and mutation (mut) on the bottom; (**B**): The gel diagram of RT-PCR transcriptional analysis. The differential bands in 293T and Hela cells were labeled a,b,c; (**C**): Minigene construction and splicing diagram; (**D**): The corresponding sequencing map to the splicing bands. Red* indicates the location of the mutation.

In order to analyzing whether the mutation c.1860G > A affects the efficient identification of its neighboring donor splicing site, we performed the splicing assay on mRNA obtained from the parent harboring the synonymous variant c.1860G > A. The wild-type(wt) and mutant Minigene were inserted into the pcMINI vector, and two recombinant vectors were transfected into HeLa, 293 T cells. A total of four samples were collected 36 h after transfection. The Minigene construction strategy of PCMINI-ITGB4-WT/MUT is to insert the complete Exon 15, Exon 16 and some intronic sequences into the pcMINI vector. The vector contained Exon A-Intron A-MSC-Intron B-Exon B. The splicing pattern of Exon A-Exon 15-Exon 16-Exon B was observed after transfection. The results of RT-PCR showed that there was an expected normal size band named Band a in the wild group of Hela and 293 T cells, and a band similar to Band a named Band b in the mutant group. There is a slightly smaller band than a, named Band c. Band a, b, and c were sequenced separately. Sequencing results showed that Band a was a normal splicing band, and its splicing pattern was Exon A-Exon 15 (99 bp)-Exon 16 (130 bp) -Exon B. Band b retained part of the Intron 15 sequence (20 BP to the right of Intron 40), and its splicing pattern was Exon A-Exon 15 (99 bp) -▽intron 15 (20 bp)- Exon 16 (130 bp) -Exon B. The deletion of the whole Exon 15 in Band c, the splicing pattern of Band c is Exon A-Exon 16 (130 BP)-Exon B. Minigene assays show that the mutation c.1860G > A (*p*. Ala 620 = ) affects the efficient identification of its neighboring donor splicing site, which results in a overall leap of Exon 15 or a partial retention of Intron 15. The results of pcMINI vector sequencing, RT-PCR, minigene construction strategy and splicing analysis are shown in Figure 4. Whether this mutation has pathogenicity still needs to be further studied.

The PGT-M involves taking some of the cells of an embryo for genetic analysis and then selecting healthy embryos for transplantation before the embryo is transferred to prevent the birth of a fetus with a genetic disease or chromosomal abnormality (16, 17). PGT-M is an effective and safe procedure for parents at high risk of transmitting genetic disorders to offspring to have children. This technique is mainly used to screen for monogenic diseases, chromosomal diseases, and aneuploidy (18–20). The PGT-M technique was used on this mother to prevent her from transferring these two heterozygous mutations of the *ITGB4* gene related to ACC to her children. This mother successfully gave birth to a healthy child.

To sum up, ACC, in addition to the different degrees of skin defects, is accompanied by ailments such as heart and limb deformities. The prognosis of ACC will be poor, possibly fatal if there are complications such as infection. Genetic factors may be one of the leading causes of ACC. The PGT-M technique is very important for families that have previously had aborted fetuses or perinatal mortality due to ACC. The PGT-M technique is valuable in reducing the incidence of ACC and improving the prognoses of newborns.

## Data Availability

The original contributions presented in the study are included in the article/Supplementary Material, further inquiries can be directed to the corresponding author/s.
